# Supplementary material for: Electrolysis of a molten semiconductor
Source: Nat Commun. 2016 Aug 24;7:12584. doi: 10.1038/ncomms12584 (PMC4999513; doi:10.1038/ncomms12584)
Supplement: Supplementary Information — Supplementary Figures 1-6, Supplementary Table 1, Supplementary Note 1 and Supplementary References. [file ncomms12584-s1.pdf]

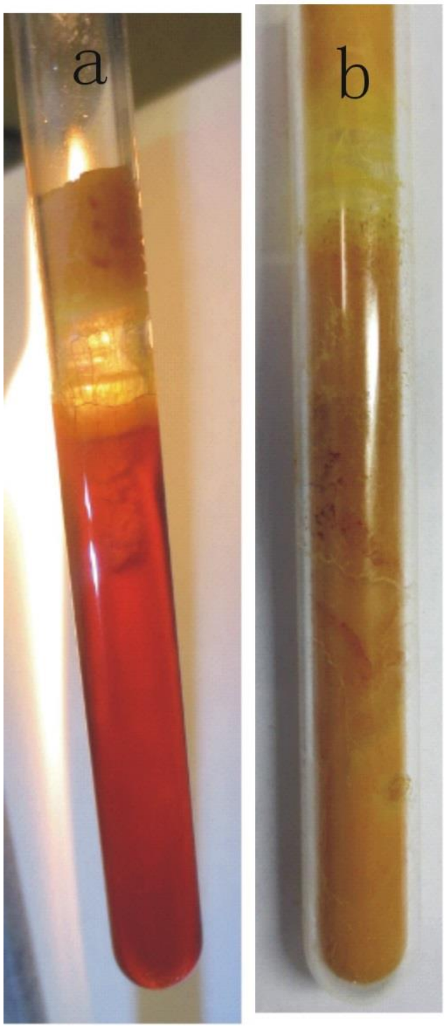

Supplementary Figure 1| Direct observation of the NaCl-KCl-Na_2S melt in a transparent quartz cell. Images of NaCl-KCl-Na_2S: in molten state (a) and solid state (b).

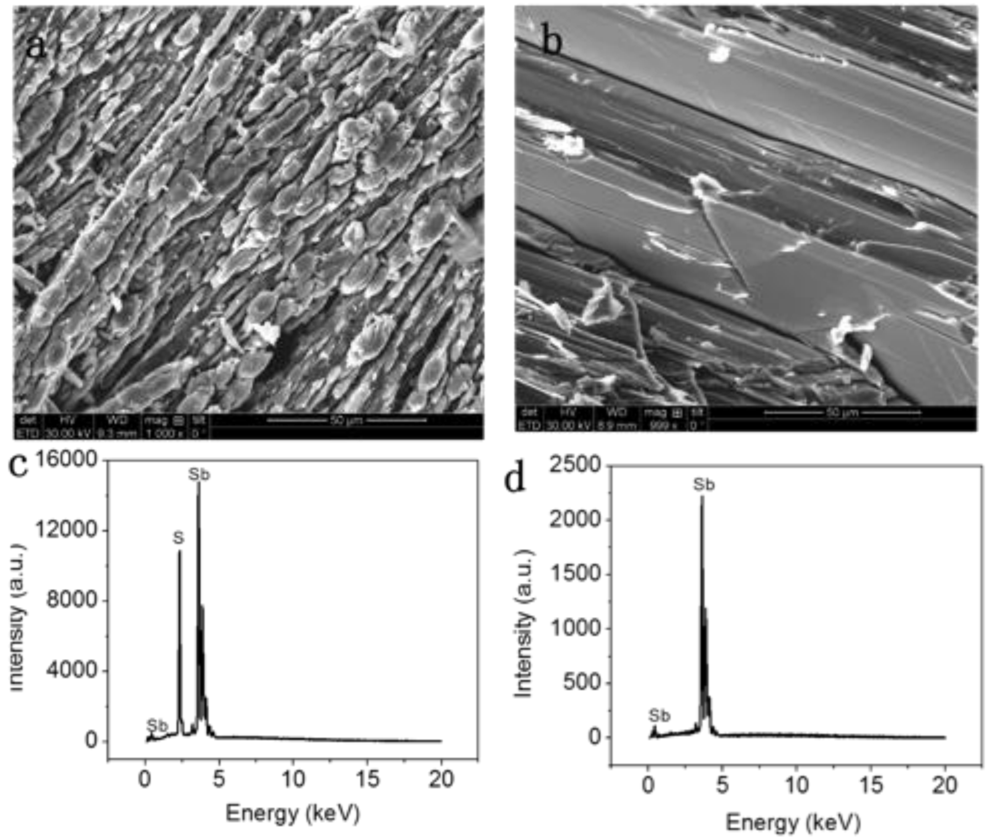

Supplementary Figure 2| SEM and EDS analysis of the cathode metal product. SEM images of Sb_2S_3 (a) and electrolytic Sb (b) obtained potentiostatically at 1.0V *vs.* Na^+/Na, and their corresponding spectra of EDS (c, d).

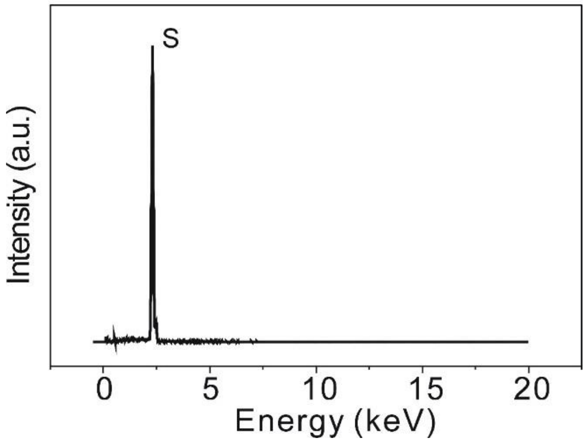

Supplementary Figure 3| EDS analysis of anode product. EDS spectrum of the yellow anode product on the side wall of the quartz cell

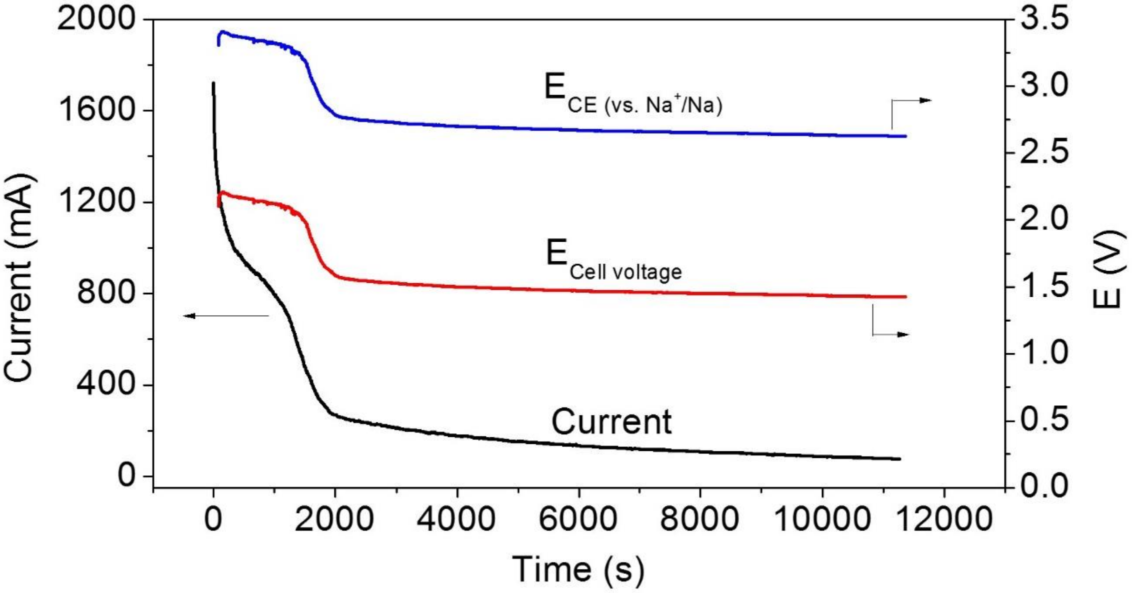

Supplementary Figure 4| Current and voltage profiles. Voltage and current profiles vs time of the graphite counter electrode and cell during potentiostatic electrolysis of Sb_2S_3 at 1.0 V *vs.* Na^+/Na.

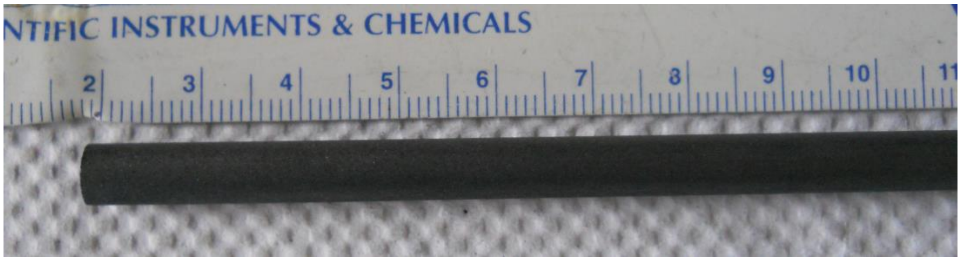

Supplementary Figure 5| Digital picture of anode. Graphite anode after one week of service in NaCl-KCl-Na_2S.

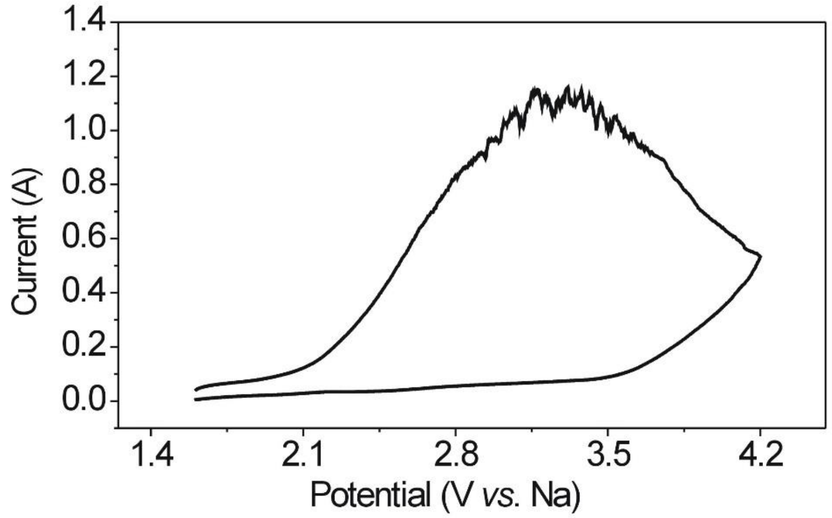

Supplementary Figure 6| Cyclic voltammogram of graphite electrode. CV of graphite working electrode in NaCl-KCl-Na_2S at 700ºC. Scan rate: 100 mV s^{-1}.

Supplementary Table 1| Thermodynamic analysis. Selected standard reduction potentials for the cathodes in molten chloride at 700°C, assuming all species to be in their standard state at unit activity (FactSage).

Reaction	E^o (V)
$Cl_2(g) + 2 e^- = 2Cl^-$	3.3
$S_2(g) + 4e^- = 2S^{2-}$	1.55
$Sb_2S_3 + 6e^- = Sb + 3 S^{2-}$	1.3
$Na^+ + e^- = Na$	0

Supplementary Note 1

Pyrometallurgy and hydrometallurgy of antimony

Antimony is a metalloid with main applications in flame-retardants, batteries and as materials additive[1]. It is found predominantly on earth as mineral stibnite (Sb_2S_3) from which elemental Sb is extracted via traditional extractive methods such as pyro-metallurgy and hydrometallurgy[2]. A first pyro-metallurgical route converts Sb_2S_3 to volatile Sb_2O_3 by roasting, which is then carbothermically reduced to Sb at temperatures exceeding 1200ºC as shown in reaction (3).

$$Sb_2O_3\ (\ell) + \frac{3}{2}\ C\ (s) = 2\ Sb\ (\ell) + \frac{3}{2}\ CO_2\ (g) \qquad (3)$$

A second pyro-metallurgical route consists of chemical reduction of molten Sb_2S_3 with scrap iron at temperatures ranging from 550 to 600ºC (eq. 4). The metal obtained is subsequently subjected to a purification step to dispose of iron and sulfur impurities[2].

$$Sb_2S_3\ (\ell) + 3\ Fe\ (s) = 2\ Sb\ (s) + 3\ FeS\ (s) \qquad (4)$$

Finally, hydrometallurgical routes usually consist of two steps, namely, leaching of the mineral and subsequent electro-winning in aqueous solution[2] and poses additional efforts for product recovery. In this manuscript, we demonstrate the method of an electron blocking secondary sulfide ion conductive electrolyte and enable Sb to be processed in a simple, clean and energy efficient manner via modern electrometallurgy.

Results of Potentiostatic Electrolysis

Potentiostatic electrolysis was employed to determine an appropriate current density to conduct galvanostatic electrolysis while avoiding secondary electrolyte decomposition (chlorine

evolution). During potentiostatic electrolysis, the potential of the working electrode was held constantly at 1V (vs Na/Na^+), the potential of the counter electrode and the cell voltage were monitored (Supplementary Fig. 4). In this experiment, features measured in the cell voltage are attributed to the half reaction at the counter electrode. At the beginning of the electrolysis, we observe that the counter electrode potential takes the value of 3.4 V (vs Na/Na^+) and the current density settles to ~1800 mA cm^{-2} (Supplementary Fig. 4). Gradually, the potential of the counter electrode decreased and was attended by a decrease of current density. After 2000 s the potential of the counter electrode dropped to 2.8 V, and simultaneously the current density dramatically decreased below 260 mA cm^{-2}, suggesting that most of the Sb_2S_3 had been converted to Sb. It is desirable to avoid high polarization at both electrodes to suppress potential side reactions such as chlorine evolution at the anode and co-deposition of sodium and / or potassium at the cathode. As shown in Supplementary Fig. 4, at a current density of ~500 mA cm^{-2}, the potential at the counter electrode floats to ~3.0 V, lower than the estimated potential for Cl_2 evolution at 3.3 V (see Supplementary Table 1), and higher than the estimated potential for sulfur evolution at 1.55V.

Supplementary References

1. Butterman, C. M. & Carlin, J. F. Mineral Commodity Profiles: Antimony. *United States Geological Survey* (2003).

2. Anderson, C.G. The metallurgy of antimony. *Chemie der Erde.* **72**, 3-8 (2012).
